# The E3 Ligase UBR5/Hyd Ensures Meiotic Fidelity Through Catalysis-Independent Regulation of β2-Tubulin in *Drosophila*

**DOI:** 10.3390/genes16111245

**Published:** 2025-10-22

**Authors:** Lin Zhou, Lang Lin, Yan Zhang, Chenghao Shen, Yun Qi, Xinhua Lin

**Affiliations:** 1 State Key Laboratory of Genetics and Development of Complex Phenotypes, School of Life Sciences, Greater Bay Area Institute of Precision Medicine (Guangzhou), Zhongshan Hospital, Fudan University, Shanghai 200433, China; lzhou17@fudan.edu.cn (L.Z.);; 2 State Key Laboratory of Eye Health, Eye Hospital, Wenzhou Medical University, Wenzhou 325027, China

**Keywords:** spermatogenesis, meiosis, β2-tubulin, UBR5, *Drosophila*

## Abstract

**Background:** Spermatogenesis depends on precise cytoskeletal regulation, particularly the microtubule system; however, the mechanisms governing tubulin homeostasis during meiosis are poorly defined. While the E3 ubiquitin ligase Hyd (Hyperplastic discs), the *Drosophila* homolog of UBR5 (Ubiquitin Protein Ligase E3 Component N-Recognin 5), plays roles in diverse cellular processes, its precise role in male meiosis is unknown. This study aims to define the function and expression dynamics of Hyd during *Drosophila* spermatogenesis and elucidate whether it acts independently of its canonical ligase activity. **Methods:** Using *Drosophila* genetics, immunofluorescence, CRISPR/Cas9-mediated tagging, and mosaic clonal analysis, we characterized Hyd expression and function in the testis. Hyd knockdown and rescue experiments were performed with wild-type and catalytically inactive transgenes. β2-tubulin expression and microtubule organization were assessed in *hyd* mutant clones. **Results**: Hyd exhibits a dynamic, stage-specific expression pattern, localizing to nuclear and meiotic structures. Hyd loss led to meiotic arrest, disrupted spindle formation, aberrant centrosome behavior, and failure of spermatid elongation. Genetic rescue demonstrated that both wild-type and catalytically inactive Hyd partially restored spermatid elongation, indicating a catalysis-independent role. Furthermore, Hyd deficiency resulted in β2-tubulin overexpression, disrupted microtubule organization, and abnormal spermatocyte morphology. **Conclusions:** Hyd ensures meiotic fidelity in *Drosophila* by fine-tuning β2-tubulin expression independently of its E3 ubiquitin ligase activity. These findings reveal a non-proteolytic function for UBR5/Hyd in cytoskeletal regulation during male gametogenesis, providing new insights into tubulin homeostasis in meiosis.

## 1. Introduction

Male infertility is a significant clinical concern, affecting approximately 7% of the global male population of reproductive age and posing substantial psychological and economic burdens on affected couples [1]. Despite its prevalence, the etiology of a considerable proportion of cases remains idiopathic, largely due to our incomplete understanding of the genetic and molecular mechanisms governing human spermatogenesis [2].

Spermatogenesis is a fundamental biological process essential for male fertility, involving tightly regulated cellular events such as spermatocyte differentiation, meiosis, and spermiogenesis [3]. These processes rely on precise cytoskeletal regulation, particularly the microtubule network, which is vital for meiotic spindle assembly, chromosome alignment, and spermatid elongation [4]. Maintaining proper tubulin stability and expression is crucial, as dysregulation can lead to impaired chromosome dynamics and male infertility [5]. However, the regulators that maintain tubulin homeostasis during spermatogenesis remain poorly characterized.

*Drosophila melanogaster* serves as a powerful and complementary model organism for dissecting the genetics of spermatogenesis [6]. Its well-conserved cellular processes and unparalleled genetic tractability—featuring a vast collection of tissue-specific drivers, mutant lines, and sophisticated tools for clonal analysis and genome editing—make it invaluable for precise mechanistic studies [7,8,9,10]. In *Drosophila* testes, germline stem cells (GSCs) and somatic cyst stem cells (CySCs) are anchored around a niche composed of hub cells. GSCs divide asymmetrically, producing one self-renewing GSC and one gonialblast (GB). Each GB is enveloped by two cyst cells derived from CySCs and undergoes four mitotic divisions to form a cyst of 16 spermatogonia. These spermatogonial cells then differentiate into mature spermatocytes, which undergo meiosis to produce 64 haploid spermatids [11,12]. After maturation and elongation, the resulting sperm are transported to the seminal vesicle for storage [13,14].

UBR5, an evolutionarily conserved HECT-family E3 ubiquitin ligase, mediates the ubiquitination and proteasomal degradation of substrates containing N-degrons [15,16]. Recognized as a tumor suppressor, UBR5 is frequently dysregulated in various human cancers and plays essential roles in diverse biological processes including DNA damage repair, transcriptional control, stem cell maintenance, and signal transduction [17,18]. In *Drosophila*, the UBR5 homolog Hyd similarly functions as a growth regulator by suppressing over-proliferation in the wing imaginal disc [18,19]. Although sterility in *hyd* mutants has been documented for decades [20], only recent evidence has revealed specific defects in spermatogenesis—including aberrant chromosome condensation, disrupted centrosome behavior, and impaired spermatid elongation [7,21,22]. However, the expression pattern of Hyd and the mechanistic basis by which it regulates spermatogenesis remain largely unknown.

Here, we demonstrate that Hyd regulates male meiosis through a catalysis-independent mechanism, by negatively modulating β2-tubulin expression to ensure proper cytoskeletal organization and faithful meiotic division. Our findings reveal a novel regulatory axis linking an E3 ubiquitin ligase to tubulin expression and meiotic cytoskeletal dynamics, providing new mechanistic insights into spermatogenesis.

## 2. Materials and Methods

### 2.1. Fly Husbandry and Stocks

*Drosophila* stocks were maintained at 25 °C under a 12/12 h light/dark cycle and 60% relative humidity on a standard cornmeal-yeast-agar medium (1% agar, 3.6% yeast, 2% yellow corn meal, 5.4% sugar, and 3% molasses). Flies were cultured in vials at a density of 20–30 individuals.

Fly stocks used in this study are either described in FlyBase or as otherwise specified. The wild-type strains used were *w^1118^* (BL5905). Other strains used were as follows: w[*]; P{w[+mC]=dj-GFP.S}AS1/CyO (BL5417), *y[1]* w[*] P{w[+mC]=His2Av.GFP(S65T)}1 P{ry[+t7.2]=hsFLP}12 P{ry[+t7.2]=neoFRT}19A/FM7a (BL32045), w[*]; P{w[+mC]=protamineB-eGFP}2/CyO (BL58406), w[*]; P{w[+mC]=UAS-mCD8.ChRFP}3 (BL27392), *y*[1] sc[*] v[1] *sev*[21]*; P{y[+t7.7] v[+t1.8]=VALIUM20-EGFP.RNAi.4}attP2* (BL41553), P{ry[+t7.2]=hsFLP}22, w[*] (BL8862), *y*[1] *w*[1118]*; P{w[+mC]=UAS-FLP.Exel}3* (BL8209), *kni[ri-1] hyd*[15] *e*[1]*/TM3, Sb*[1] (BL3718), *w*[1118]*; P{ry[+t7.2]=neoFRT}82B P{w[+mC]=Ubi-mRFP.nls}3R* (BL30555), *y*[1] sc[*] v[1] *sev*[21]*; P{y[+t7.7] v[+t1.8]=TRiP.HMS00343}attP2* (BL32352), and *y*[1] *v*[1]*; P{y[+t7.7] v[+t1.8]=UAS-LUC.VALIUM10}attP2* (BL35788) were obtained from the Bloomington Drosophila Stock Center (BDSC). *Bam-Gal4-VP16* [23], *Vasa-EGFP* [24], and *β2-tubulin-GFP* [25] were kindly provided by Dr. Zhaohui Wang.

The transgenic flies, *UAS-V5-hyd* and *UAS-V5-hydC2854S*, are generated according to site-directed integration protocol and used for rescue analysis. Briefly, Hyd cDNA (WT) and the CS mutant were subcloned into pUAST-attB-V5 and inserted into the attP40 site according to standard protocol. V5-tagged Hyd-CS, which displays a mutated HECT domain by conversion of the conserved catalytic cysteine at position 2854 to serine. The construction, validation and functional reliability of this catalytically inactive *UAS-V5-hydC2854S* construct have been described in previous studies [26,27]. All key transgenic lines, including the RNAi lines and the *hyd^15^* allele, were backcrossed into the *w^1118^* background for at least three generations to homogenize the genetic background.

### 2.2. Immunofluorescence Staining and Microscopy

Immunohistochemistry was performed on testes dissected from flies at specified days post-eclosion. Control and experiments were aged and tested at the same time. Adult flies were anesthetized using a CO_2_ pad, and testes with desired genotype were dissected in 1× phosphate-buffered saline (PBS) and fixed with 4% paraformaldehyde (PFA) in PBS for 30 min. After fixation, samples were washed twice with PBS (10 min per wash) and permeabilized with 0.1% Triton X-100 in PBS (PBSTx) for 1 h at room temperature. Following permeabilization, samples were blocked with blocking solution (5% fetal bovine serum in PBSTx) for 1 h and then incubated with primary antibody diluted in the blocking solution overnight at 4 °C. After three 15 min washes with PBS at room temperature, samples were incubated with secondary antibody diluted in the blocking solution for 2 h at 25 °C in the dark. Finally, samples were washed three times with PBSTx (15 min each) and mounted using VECTASHIELD^®^ PLUS Antifade Mounting Medium with DAPI (Catalog #: H-2000-10; Vector Laboratories, Burlingame, CA, USA). Antibodies used as follows: mouse anti-Fibrillarin (1:200; Catalog #: T5326-25UL; Sigma-Aldrich, St. Louis, MO, USA), donkey anti-rabbit Alexa Fluor^®^ 488 (1:800; Catalog #: A32790; Invitrogen, Waltham, MA, USA), goat anti-rabbit Alexa Fluor^®^ 568 (1:800; Catalog #: A-11036; Invitrogen, Waltham, MA, USA), donkey anti-mouse Alexa Fluor^®^ 568 (1:800; Catalog #: A10037; Invitrogen, Waltham, MA, USA), and rabbit anti-Hyd (1:2000; [26]). The anti-Hyd antibody is a well-characterized reagent from our previous publication [26], where it was generated in rabbit using a purified GST-Hyd (aa 570–670) antigen and its specificity was thoroughly validated.

All immunofluorescence images were obtained using an Olympus FV3000 Laser Scanning confocal microscope (Olympus, Tokyo, Japan) with the same parameter settings. Confocal images were acquired with a 60× oil immersion objective (NA 1.4) at a resolution of 1024 × 1024 pixels. Scanning was performed in a unidirectional galvano mode line-sequentially. The following settings were used for each channel: DAPI (PMT voltage: 478 V; pinhole: 180 μm), Alexa Fluor 488 (PMT voltage: 395 V; pinhole: 200 μm), Alexa Fluor 568 (PMT voltage: 420 V; pinhole: 200 μm), and Alexa Fluor 647 (PMT voltage: 450 V; pinhole: 220 μm). Original .oib files were exported as TIFF files using the FV31S-DT (Olympus) software. Final figures were assembled using Adobe Illustrator (version 2025), and image sizes were uniformly adjusted in Adobe Photoshop (version 2022) to meet journal requirements.

For the quantification of Hyd staining intensity, all confocal images within a given experiment were acquired under identical imaging parameters (laser power, gain, and exposure time) to ensure comparability. Regions of interest (ROIs) were manually delineated around individual cells, which were identified at different spermatogenic stages based on the morphology revealed by β2-tubulin-GFP and DAPI nuclear staining. Background fluorescence, determined as the mean intensity from three cell-devoid regions in each image, was subtracted from the mean intensity of every cellular ROI. All quantifications were performed in a blinded manner to prevent bias, with the analyst unaware of the experimental group assignments until the entire analysis was completed.

### 2.3. Mosaic Clonal Analysis

The *hyd^15^* allele is a null allele characterized by a nonsense mutation that introduces a premature stop codon at tryptophan 485 (aa485W>STOP) [26,28]. Homozygous *hyd^15^* mutant clones were generated in a heterozygous background using FLP-mediated mitotic recombination [29]. Briefly, virgin females of the genotype *hs-flp;; FRT82B, Ubi-mRFP* were crossed to males carrying either *FRT82B* or *FRT82B,hyd^15^/TM6B*. For each cross, 20 virgin females and 5 males were placed per vial. F1 male progeny lacking the TM6B balancer were collected within 2–3 days after eclosion. These males were subjected to a 2 h heat shock in a 37 °C water bath to induce FLP/FRT recombination and then maintained at 25 °C. Testes were dissected at 5, 7, and 10 days after heat-shock (AHS) and imaged using an Olympus FV3000 confocal microscope. These time points were chosen to allow for the development and clear visualization of mutant clones in germline cells at distinct stages, including the spermatocyte, meiotic divisions, and late spermatid bundle, based on the known duration of *Drosophila* spermatogenesis (approximately 10–11 days at 25 °C) [30,31].

The control genotype was *hs-flp/Y; β2-tubulin-GFP/+; FRT82B,Ubi-mRFP/FRT82B*, and the experimental genotype was *hs-flp/Y; β2-tubulin-GFP/+; FRT82B,Ubi-mRFP/FRT82B,hyd^15^*. Control flies were subjected to the identical heat-shock regime and analysis to account for any background recombination or non-specific effects of heat-shock. For each genotype and time point, a minimum of 20 clones were scored from at least 10 individual testes derived from three independent biological replicates (heat-shock events). Clones were identified by the absence of RFP fluorescence and scored as positive only if they contained a clearly identifiable spermatocyte or later-stage germ cells. All clone imaging was performed using the same Olympus FV3000 confocal microscope and that images for quantitative comparison were acquired with identical settings to ensure consistency and comparability.

### 2.4. Genomic Editing by CRISPR-Cas9

GFP-Hyd tagin line was generated as described previously [32]. Briefly, a single-guide RNA (sgRNA) targeting the first intron of *hyd* was designed and cloned into the pU6-BbsI-chiRNA vector (Addgene #45946). The sgRNA sequence is 5′-caattacgcacatgtgaacgcgg-3′. The PAM sequence is cgg and is located on the sense strand. Potential off-target sites were predicted using CRISPRdirect [33], available at https://crispr.dbcls.jp/ (accessed on 23 October 2023). We performed Sanger sequencing of the top potential off-target sites in our final GFP-Hyd knock-in line and confirmed that no off-target mutations occurred. Homology arms flanking the insertion site were amplified and cloned into the pHD-DsRed-attP donor vector [32,34]. The donor plasmid was designed with 1199 bp and 1432 bp left and right homology arms, respectively. The sfGFP cassette was inserted in-frame, immediately downstream of the start codon (ATG) of the first exon of the *hyd* gene, ensuring the expression of an N-terminal sfGFP-tagged Hyd fusion protein. The complete plasmid sequence (Snapgene file) and all primer sequences used for plasmid construction and verification have been deposited in the Supplementary Data.

Embryo microinjection was performed by the *Drosophila* Resource and Technology Platform at the Shanghai Institute of Biochemistry and Cell Biology, Chinese Academy of Sciences. A mixture of the donor plasmid (200 ng/μL) and the sgRNA plasmid (100 ng/μL) was co-injected into embryos of the genotype *y*[1] M{w[+mC]=nanos-Cas9.P}ZH-2A w[*] (BL54591) [34,35]. A total of 100 embryos were injected, resulting in a survival rate of 78%. All P0 flies were crossed with balancer stock (genotype: *if/Cyo; MKRS,Sb/Tm6B*). The desired knock-in line was identified by PCR amplification followed by Sanger sequencing from P1 flies. To homogenize the genetic background, the established line was backcrossed for 3 times with the balancer stock.

### 2.5. Statistical Analyses

Data are representative of at least three independent biological replicates in figures. Statistical analysis of the relative staining intensity of Hyd across spermatogenic stages was performed on confocal images from wild-type testes using Fiji ImageJ (version 1.54f) [36] and GraphPad Prism (version 10.0). Data, normalized to the mean fluorescence intensity of GSCs, are presented as the mean ± SEM from at least three independent experiments. The statistical unit was defined as a single cell, with 20 cells analyzed per stage from each biological replicate. The Shapiro–Wilk and Levene’s tests were used to verify the assumptions of normality and homogeneity of variances, respectively. Based on the outcome, comparisons between two groups were made using a two-tailed Student’s *t*-test, while comparisons among three or more groups were analyzed by one-way ANOVA followed by Tukey’s multiple comparisons test. A *p*-value < 0.05 was considered statistically significant (* *p* < 0.05, ** *p* < 0.01, *** *p* < 0.001, n.s., not significant).

## 3. Results

### 3.1. Hyd Displays a Dynamic, Stage-Specific Expression Pattern During Spermatogenesis

**Figure 1:**
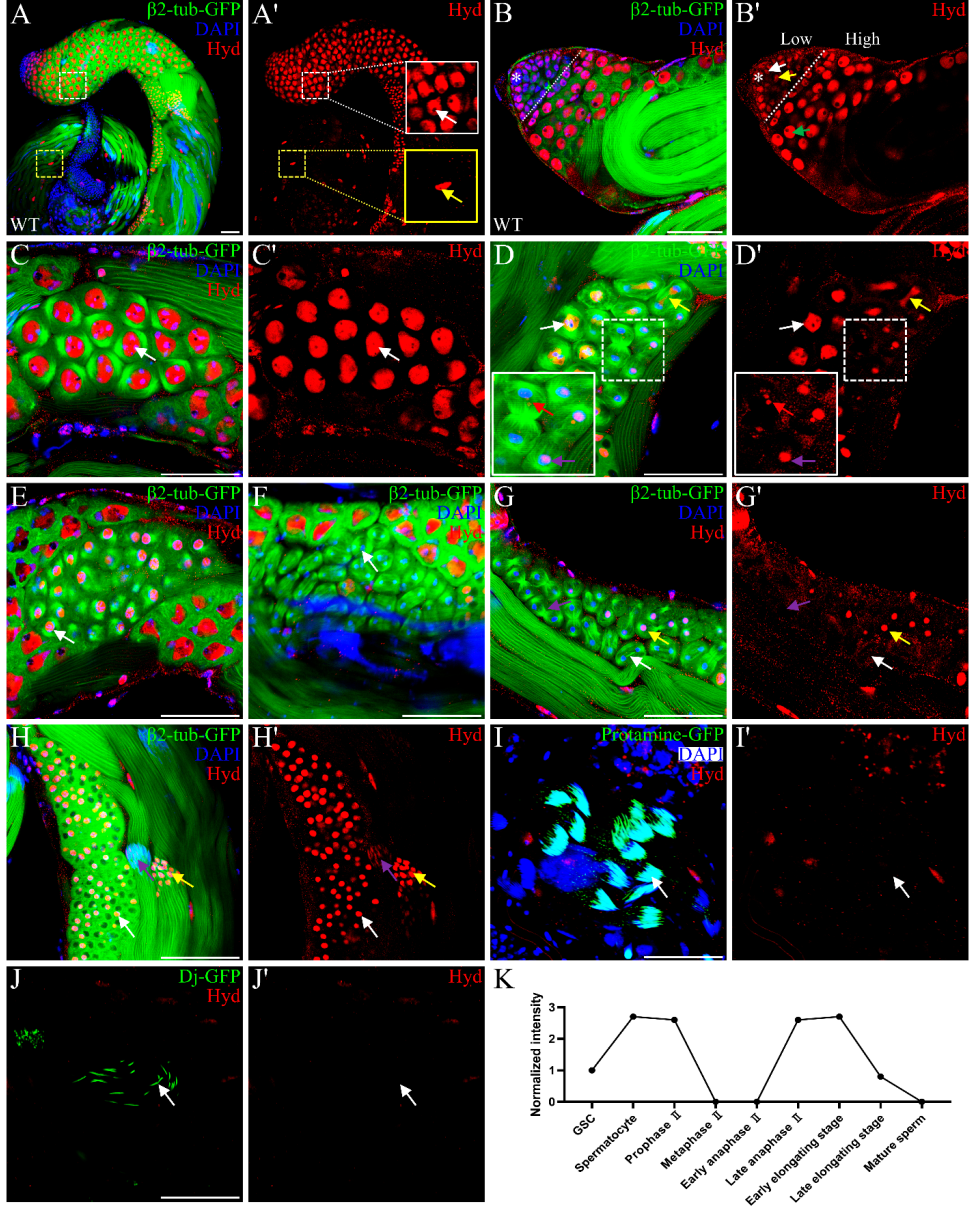
Dynamic and stage-specific expression of Hyd during spermatogenesis. (**A**,**A’**) Immunofluorescence staining of Hyd (red) in *Drosophila* testes. Hyd localizes internally to β2-tubulin–GFP-labeled germ cells (white dashed box). Pore-like gaps in Hyd signal are visible in germ cells (white arrow) and late cyst cells (yellow arrow). (**B**,**B’**) Differential Hyd expression at the testis apex: low in the germinal proliferation center (left, dashed outline), including the hub (asterisk), GSC (white arrow), and GB (yellow arrow); high in early spermatocytes (green arrow). (**C**–**E**) Hyd persists through meiosis I and into prophase II. Specific stages are indicated: prophase I (**C**, white arrow), metaphase I (**D**, white arrow), early anaphase I (**D**, yellow arrow), late anaphase I (**D**, red arrow), telophase I (**D**, purple arrow), and prophase II (**E**, white arrow). (**F**–**G’**) Hyd is absent from metaphase II to early anaphase II (**F**–**G’**, white arrows) and reaccumulates by late anaphase II (**G**,**G’**, yellow arrows). (**H**–**J’**) Post-meiotic expression of Hyd in spermatids at successive developmental stages: round spermatids (**H**,**H’**, white arrow), early elongation (**H**,**H’**, yellow arrow), late elongation (**H**,**H’**, purple arrow), the final stage of elongation (**I**,**I’**, white arrow; marked by Protamine-GFP), and mature spermatids (**J**,**J’**, white arrow; marked by Dj-GFP). The arrow in Figure 1I’ indicates the disappearance of Hyd signal in Protamine-GFP-positive final elongated spermatids, a stage marked by the completion of histone-to-protamine transition. The arrow in Figure 1J’ points to the absence of Hyd expression in mature sperm within the seminal vesicle, which are labeled by Dj-GFP. (**K**) Schematic summarizing the dynamic expression pattern of Hyd throughout spermatogenesis. The x-axis represents distinct stages of spermatogenesis, and the y-axis shows the relative mean fluorescence intensity of Hyd (normalized to the intensity in GSCs; n = 20 cells per stage). Scale bars: 50 μm. Data are representative of at least three independent biological replicates. Sample sizes (n) for each panel are as follows: (**A**–**J**) n ≥ 20 testes.

To investigate the function of Hyd in *Drosophila* spermatogenesis, we first examined its expression in the testis. Immunofluorescence staining with an anti-Hyd antibody revealed widespread Hyd expression throughout the testis (Figure 1A,A’). In germ cells labeled with β2-tubulin-GFP, Hyd signal was complementary to and encased within the β2-tubulin-GFP (Figure 1A,C), implying a potential spatial or functional relationship between Hyd and the microtubule cytoskeleton. Additionally, small pore-like gaps were observed in Hyd staining in both germ cells and late cyst cells (Figure 1A,A’, white and yellow boxes, respectively). Using H2AV-GFP to mark nuclei and anti-Fibrillarin to label nucleoli, we observed Hyd co-localized with H2AV-GFP (Figure S1A–A”) but was excluded from Fibrillarin-positive regions (Figure S1B–C”), demonstrating that Hyd localizes to the nucleus but is absent from nucleoli.

We next analyzed the dynamic expression pattern of Hyd throughout spermatogenesis. Meiotic stages were classified according to established cytological criteria based on nuclear and microtubule morphology [37,38]. Hyd expression was relatively low in GSCs and early spermatogonia (Figure 1B,B’, white and yellow arrows, respectively). Its level increased markedly in early spermatocytes (Figure 1B,B’, green arrow) and remained elevated throughout meiotic prophase I (Figure 1C,C’, white arrow). During metaphase I, Hyd was localized to the equatorial plate but excluded from condensed DAPI-stained chromosomes (Figure 1D,D’, white arrow). As anaphase I commenced, Hyd migrated toward both spindle poles in concert with segregating chromosomes (Figure 1D,D’, yellow arrow). By late anaphase I, it had condensed into two or three distinct puncta per daughter cell (Figure 1D,D’, red arrow). These puncta subsequently coalesced into spherical structures during telophase I (Figure 1D,D’, purple arrow), which persisted into prophase II (Figure 1E, white arrow). Interestingly, Hyd expression disappeared abruptly at metaphase II (Figure 1F, white arrow) and remained undetectable during early anaphase II (Figure 1G,G’, white arrow) but reappeared at high levels by late anaphase II (Figure 1G,G’, yellow arrow).

After meiosis, 64 round spermatids were formed. Hyd remained highly expressed and was enriched within the nucleus at the onion stage (Figure 1H,H’, white arrow). As nuclear elongation commenced, Hyd expression gradually decreased (Figure 1H,H’, yellow arrow, early elongation; purple arrow, late elongation) and was eventually undetectable by the end of elongation, as marked by Protamine-GFP labeling [39] (Figure 1I,I’, white arrow). Finally, Hyd was absent in mature sperm, visualized by Dj-GFP signal [40] (Figure 1J,J’, white arrow). In summary, Hyd is widely expressed in the *Drosophila* testis and exhibits a dynamic, stage-specific expression pattern throughout spermatogenesis (Figure 1K).

### 3.2. Hyd Depletion Disrupts Meiotic Progression and Spermatid Differentiation

**Figure 2:**
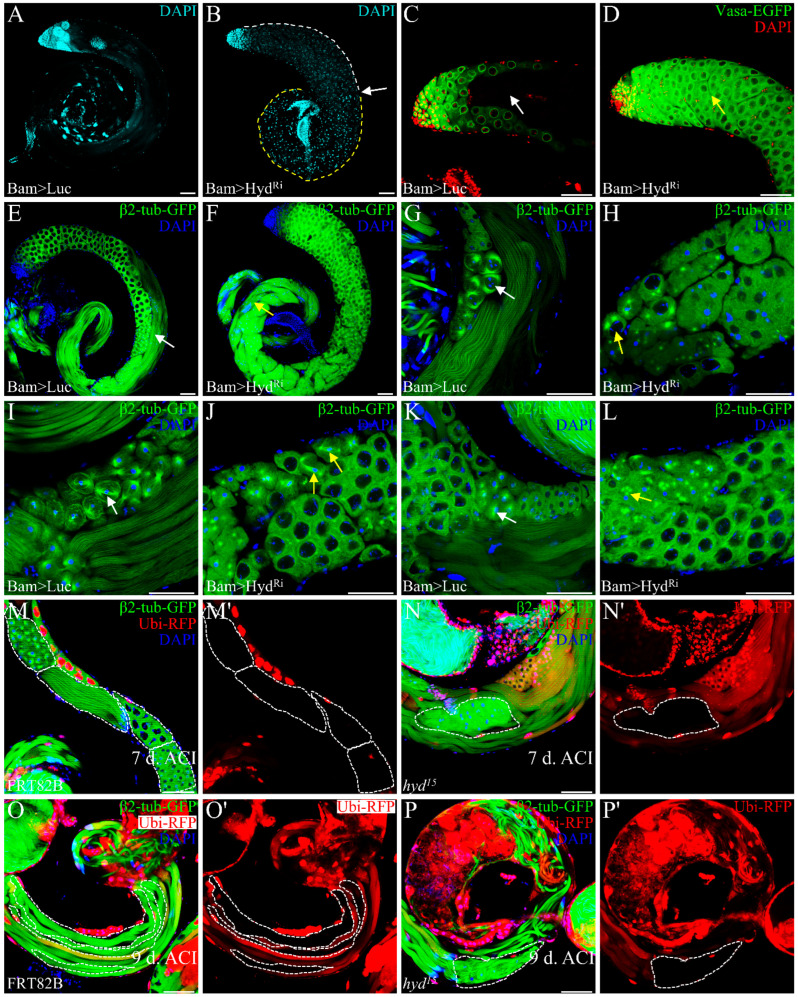
Hyd is required for meiotic progression and germ cell differentiation. (**A**,**B**) DAPI staining of Hyd knockdown testis shows accumulation of spermatocytes (white dashed line) and aberrant punctate nuclei (yellow dashed line), with a clear boundary (white arrow). (**C**,**D**) Vasa-EGFP labeling in control (**C**) and Hyd knockdown (**D**) testes. Note loss of Vasa-EGFP-negative voids (**C**, white arrow) and presence of Vasa-EGFP-positive spermatocyte clusters (**D**, yellow arrow). (**E**,**F**) β2-tubulin-GFP expression in control (**E**) and Hyd knockdown (**F**) testes, showing organized elongated spermatid bundles (**E**, white arrow) versus disorganized bundles (**F**, yellow arrow). (**G**–**L**) Meiotic defects in Hyd knockdown testes. Compared to controls (**G**,**I**,**K**; white arrows), Hyd knockdown causes impaired chromatin condensation and centrosome migration in prophase I (**H**, yellow arrow), abnormal metaphase I spindles (**J**, yellow arrows), and aberrant two centrosome pairing in prophase II (**L**, yellow arrow). (**M**–**N’**) *hyd^15^* mosaic clones 7 days after induction. Control clones show normal meiotic and post-meiotic cysts (**M**, white dashed line), arranged in apical-to-basal order: meiosis II, elongating spermatids, spermatocytes, and round spermatids. Mutant clones contain cysts with reduced cell number and abnormal round spermatids (**N**, white dashed line). (**O**–**P’**) *hyd^15^* mosaic clones 9 days after induction. Control clones display organized spermatid bundles (**O**, white dashed line), whereas mutant clones exhibit defective spermatid elongation (**P**, white dashed line). Scale bars: 50 μm. Data are representative of at least three independent biological replicates. Sample sizes (n) for each panel are as follows: (**A**–**D**) n = 20 testes; (**E**–**L**) n = 10 testes; (**M**–**P’**) n ≥ 20 clones per time point.

To further investigate the function of Hyd in germ cells, we performed Hyd knockdown specifically during the 4–16 cell stages by *Bam-Gal4-VP16* [13]. Depletion of Hyd driven by *Bam-Gal4-VP16* results in a significant reduction in Hyd expression, confirming the efficiency of the Hyd-RNAi line (Figure S2). Although testis size was unaffected, fertility assays revealed complete sterility. DAPI staining revealed an accumulation of spermatocytes in the upper testis region (Figure 2B, white dashed line), while the lower region was filled with brightly stained punctate nuclei (Figure 2B, yellow dashed line), with a clear demarcation between the two regions (Figure 2B, white arrow), suggesting disrupted germ cell differentiation. Consistently, using *Vasa-EGFP* transgenes to label germline, In the control testes, characteristic Vasa-EGFP-negative voids, which accommodate elongating spermatid bundles, were readily apparent (Figure 2C, white arrow). However, upon Hyd knockdown, these voids were conspicuously absent. Instead, the corresponding regions were filled with clusters of Vasa-EGFP-positive spermatocytes (Figure 2D, yellow arrow). This phenotype indicates a failure in germ cell differentiation beyond the spermatocyte stage. Furthermore, Dj-GFP labeling confirmed the absence of both elongated spermatid bundles and mature sperms in Hyd knockdown testes (Figure S3), reinforcing the conclusion that germ cell differentiation is arrested.

We hypothesized that Hyd deficiency severely perturbs meiosis, thereby preventing spermatocytes from completing normal meiotic division and forming round spermatids and subsequent spermatid bundles. Using β2-tubulin-GFP to monitor meiotic progression, we observed tightly organized bundles of elongating spermatids in the middle of control testes (Figure 2E, white arrow). In contrast, Hyd knockdown testes exhibited disorganized and aberrant spermatid bundles in basal region (Figure 2F, yellow arrow). Further examination revealed multiple meiotic defects: while control testes displayed distinct meiotic stages including prophase I (Figure 2G, white arrow), metaphase I (Figure 2I, white arrow), and prophase II (Figure 2K, white arrow), these processes were severely impaired upon Hyd knockdown. Defects included impaired chromatin condensation and aberrant centrosome migration during prophase I (Figure 2H, yellow arrow), as well as abnormal spindle morphology at metaphase I (Figure 2J, yellow arrow). Additionally, prophase II frequently exhibited abnormal centrosome behavior, such as two centrosome pairs adjacent to a single nucleus (Figure 2L, yellow arrow), suggesting incomplete cytokinesis. Together, these results demonstrate that loss of Hyd disrupts key meiotic events, leading to a blockage in germ cell differentiation.

To better trace germ cell fate following Hyd loss, we generated *hyd^15^* mosaic clones. At day 7 post-induction, mutant clones contained abnormal round spermatid-like cells with heterogeneous nuclei and significantly reduced cell numbers per cyst (Figure 2N, white dashed line), contrasting with the distinct meiotic and post-meiotic clones observed in controls (Figure 2M, white dashed line). By day 9, control clones showed organized bundles of 64 elongated spermatids (Figure 2O, white dashed line), while *hyd^15^* clones exhibited disorganized spermatid bundles (Figure 2P, white dashed line), indicating defects during elongation. These results demonstrate that Hyd is essential for both round spermatid formation and spermatid elongation.

### 3.3. Hyd Regulates Germ Cell Differentiation Independently of Its E3 Ubiquitin Ligase Activity

Beyond its canonical E3 ubiquitin ligase functions, UBR5/Hyd also operates through catalysis-independent mechanisms [41]. To investigate whether Hyd maintains germ cell differentiation depends on its E3 ligase activity, we generated a GFP-tagged *hyd* allele using CRISPR/Cas9-mediated knock-in [32], in which the first exon of hyd and sfGFP were flanked by FRT sites to allow for FLP recombinase-mediated excision (Figure S4). Homozygous *GFP-Hyd* flies were viable and fertile. We next induced Flippase (FLP) recombinase expression during 4–16 cell germlines under the control of *Bam-Gal4-VP16* (Figure S5A–A”) to excise the first exon of *hyd*, thereby introducing a frameshift mutation expected to disrupt Hyd function. While sfGFP was successfully removed, Hyd protein persisted in spermatogonia (Figure S5B–C”), and flies remained fertile with normal sperm storage, indicating that the first exon is non-essential, potentially due to alternative translation initiation. Since FLP expression did not fully abolish Hyd function, we used *GFP-RNAi* to knock down both GFP and Hyd in the *GFP-Hyd* background. This approach efficiently reduced target expression (Figure S5D–D”).

**Figure 3:**
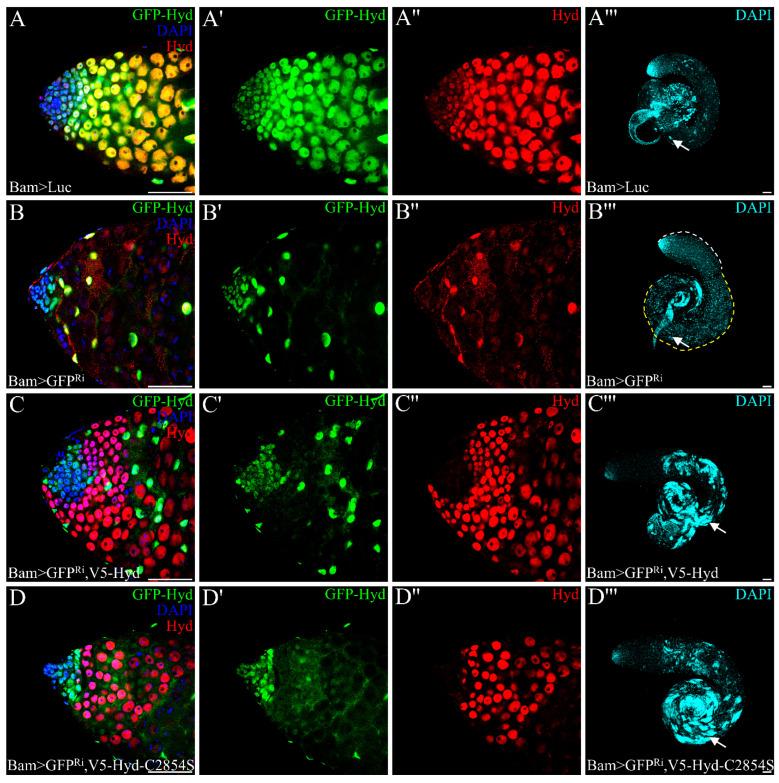
Hyd regulates germ cell differentiation independently of its E3 ubiquitin ligase activity. (**A**–**B”’**) Knockdown of Hyd via *Bam>GFP^Ri^* depletes both sfGFP (**B’**) and Hyd (**B”**) and completely blocks germ cell differentiation (**B**), compared to control testes (**A**–**A”’**). (**C**–**D”’**) Rescue experiments showed that both full-length Hyd and an E3 ligase-dead mutant partially restored spermatid elongation indicated by the presence of clustered nuclear staining representing elongated spermatid nuclei (white arrows). Scale bars: 50 μm. Data are representative of at least three independent biological replicates. Sample sizes (n) for each panel are as follows: (**A**–**A”’**) n = 15 testes; (**B**–**B”’**) n = 20 testes; (**C**–**C”’**) n = 20 testes; (**D**–**D”’**) n = 20 testes.

Thus, by means of *Bam-Gal4-VP16* to drive *GFP-RNAi* in the *GFP-Hyd* homozygous background, we observed both sfGFP and Hyd expression effectively suppressed (Figure 3B–B”), resulting in a complete block in germ cell differentiation and loss of elongating spermatids, phenocopying *Bam>Hyd^Ri^* testes (Figure 3B’’’). Rescue experiments showed that both full-length Hyd and an E3 ligase-dead mutant [26] partially restored spermatid elongation, as indicated by the presence of clustered nuclear staining representing elongated spermatid nuclei (Figure 3C–D’’’). Although the incomplete rescue may be attributed to factors such as suboptimal expression timing of the rescue transgene or persistent RNAi effects, the equivalent rescuing ability of both Hyd variants demonstrates that Hyd functions independently of its catalytic activity to support meiosis and spermatid differentiation.

### 3.4. Hyd Deletion Leads to Aberrant Accumulation of β2-Tubulin

**Figure 4:**
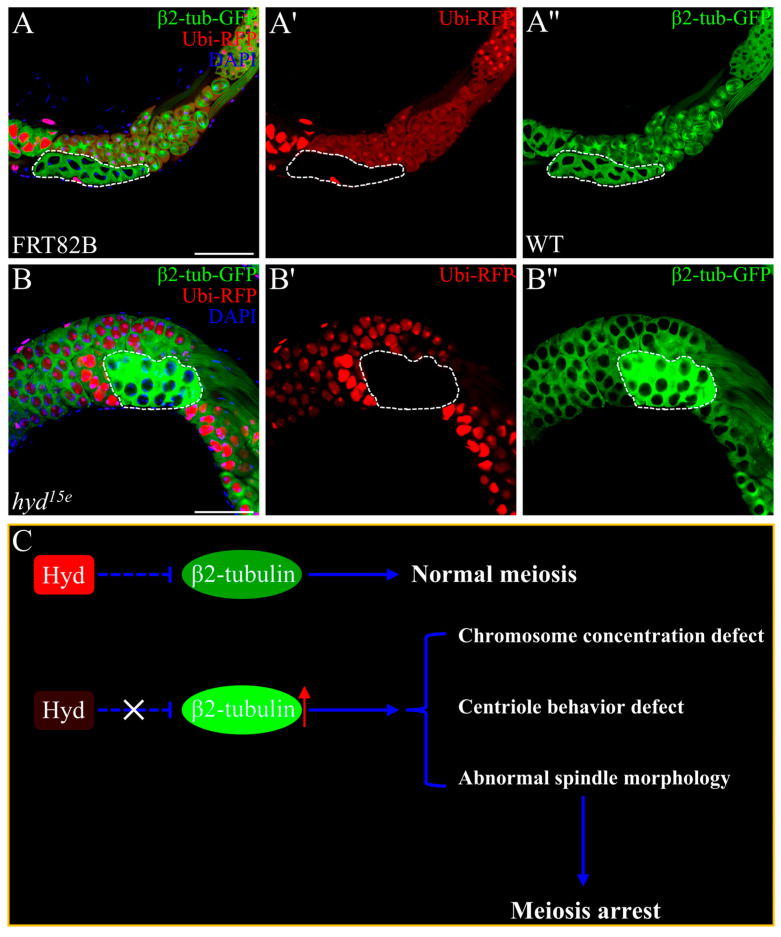
Hyd regulates spermatocyte differentiation through modulating β2-tubulin expression and cytoskeletal organization. (**A**–**A’**) Control spermatocyte clones, marked by the absence of RFP, exhibit an irregular flattened morphology and normal levels of β2-tubulin–GFP expression. (**B**–**B’**) In contrast, *hyd^15^* mutant clones show upregulated β2-tubulin–GFP signal and adopt a spherical shape, indicating disrupted cytoskeletal organization. (**C**) Proposed model: Hyd ensures proper meiotic progression by fine-tuning β2-tubulin levels and maintaining cytoskeletal architecture; loss of Hyd function leads to tubulin misregulation and subsequent meiotic arrest. The "X" denotes disruption or inhibition. The dashed line with an adjacent vertical bar represents a potential indirect inhibitory mechanism. Scale bars: 50 µm (**A**,**B**). Data are representative of at least three independent biological replicates. Sample sizes (n) for each panel are as follows: (**A**–**A”**) n = 20 testes; (**B**–**B”**) n = 20 testes.

β2-tubulin, a testis-specific microtubule protein in *Drosophila*, is essential for spermatocyte differentiation and serves critical roles in meiotic spindle assembly, nuclear shaping, axoneme formation, and spermatid elongation [4,42]. The complementary spatial relationship between Hyd and β2-tubulin expression prompted us to investigate a potential functional interplay between them. We then generated *hyd* mutant spermatocyte mosaics clones. Strikingly, Hyd loss resulted in a substantial upregulation of β2-tubulin–GFP expression within 16-cell spermatocyte cysts relative to adjacent wild-type cells (Figure 4B–B”). Moreover, late-stage hyd mutant spermatocytes exhibited a regular spherical morphology (Figure 4B–B”), in sharp contrast to the irregular flattened shape of control cells (Figure 4A–A”). These findings indicate that Hyd deficiency causes mis-regulation of β2-tubulin and disrupts cytoskeletal organization. Collectively, our data support a model in which Hyd facilitates spermatocyte differentiation by fine-tuning β2-tubulin expression. In the absence of Hyd, aberrant tubulin accumulation may impair cytoskeletal remodeling, leading to meiotic defects and subsequent arrest (Figure 4C).

## 4. Discussion

Our study identifies the E3 ubiquitin ligase Hyd as an essential regulator of meiotic progression and spermatid differentiation in *Drosophila*. Loss of Hyd leads to meiotic arrest and male sterility, and we uncover a novel, catalysis-independent function for Hyd in controlling meiotic division via regulation of β2-tubulin.

Hyd exhibits a tightly regulated spatiotemporal expression pattern, localizing predominantly to the nucleus and displaying stage-specific accumulation during meiosis and early spermiogenesis. This expression profile suggests roles in nuclear events such as chromatin organization or meiotic chromosome dynamics, consistent with the chromatin condensation defects and aberrant centrosome behavior observed in *hyd^15^/hyd^C017^* mutant [21]. The absence of Hyd disrupts multiple meiotic processes, including spindle assembly, centrosome behavior, and cytokinesis, ultimately preventing the formation of haploid spermatids. Notably, the precise localization of Hyd to the equatorial plate during metaphase I and its poleward migration during anaphase I further suggest a direct, albeit unknown, involvement in chromosome segregation or spindle dynamics. Interestingly, the discontinuous expression pattern of Hyd during meiosis II implies a potential regulatory function unique to this division stage, warranting further investigation into its biological significance.

A key and unexpected finding is that Hyd operates independently of its E3 ubiquitin ligase activity. Both full-length Hyd and a catalytically inactive E3 mutant were sufficient to partially rescue the meiotic arrest phenotype, indicating that E3 catalytic activity is dispensable for this role. This suggests that Hyd may act primarily as a structural or transcriptional regulator rather than as a canonical ubiquitin transfer enzyme. This aligns with emerging evidence that UBR5 can serve as a scaffold protein or transcriptional co-regulator, independent of its enzymatic activity [41,43]. Our data place Hyd within this broader paradigm, indicating that E3 ligases can exert profound developmental control via non-proteolytic mechanisms.

Mechanistically, we demonstrate that Hyd constrains the expression of β2-tubulin, a testis-specific tubulin isoform critical for spermatocyte differentiation, spindle dynamics, and spermatid elongation [4,44]. In the absence of Hyd, β2-tubulin is accumulated, leading to microtubule mis-regulation and aberrant spermatocyte morphology. Proper tubulin homeostasis is known to be crucial for accurate meiotic chromosome segregation and cytoskeletal remodeling during spermatogenesis [5]. Thus, Hyd acts upstream of a tubulin regulatory axis, ensuring the precise dosage of β2-tubulin required for cytoskeletal stability and meiotic fidelity.

A primary limitation of the current study is that the precise molecular mechanism by which Hyd regulates β2-tubulin levels—whether through direct interaction, transcriptional control, or an indirect pathway—remains to be fully elucidated. Given that Hyd contains a unique PABC domain, a module typically found in poly(A)-binding proteins (PABPs) that binds the mRNA poly(A) tail and regulates translation initiation and mRNA stability [45,46]—we postulate a compelling model whereby Hyd may post-transcriptionally control β2-tubulin expression. Future work should prioritize testing this hypothesis and identifying the direct mRNA or protein partners of Hyd, which will be crucial for validating the conservation of this regulatory axis in mammalian models.

## 5. Conclusions

In summary, this study reveals a catalysis-independent function for the E3 ubiquitin ligase Hyd in ensuring meiotic fidelity and spermatid differentiation in *Drosophila*, primarily through the restraint of β2-tubulin accumulation. This finding expands the functional repertoire of E3 ligases in development, highlighting that they can exert essential, profound control through non-canonical, non-proteolytic mechanisms. The conservation of UBR5/Hyd across species, coupled with the fundamental importance of cytoskeletal regulation in spermatogenesis, suggests that the mechanistic insights gained here may have direct relevance to understanding the genetic and molecular underpinnings of certain forms of idiopathic male infertility.

## Data Availability

The data supporting the findings of this study are available within the article.

## Supplementary Materials

The following supporting information can be downloaded at: https://www.mdpi.com/article/10.3390/genes16111245/s1, Figure S1: Subcellular localization of Hyd in *Drosophila* testes; Figure S2: Hyd knockdown efficiently depletes Hyd protein in the testis; Figure S3: Hyd knockdown depletes elongated spermatids and mature sperm. Figure S4: Strategy for CRISPR/Cas9-mediated generation of the *GFP-Hyd* knock-in allele; Figure S5: Hyd inactivation by FLP excision and *GFP-RNAi* knockdown; Table S1: Primer sequences used for plasmid construction and verification. Snapgene file: Complete sequence of the GFP-Hyd donor plasmid construct.
