# Supplementary material for: The E3 Ligase UBR5/Hyd Ensures Meiotic Fidelity Through Catalysis-Independent Regulation of β2-Tubulin in Drosophila
Source: Genes (Basel). 2025 Oct 22;16(11):1245. doi: 10.3390/genes16111245 (PMC12652988; doi:10.3390/genes16111245)
Supplement: Supplementary file 1 [file genes-16-01245-s001.zip › Supplementary Data - revise.pdf]

Supplementary Figures

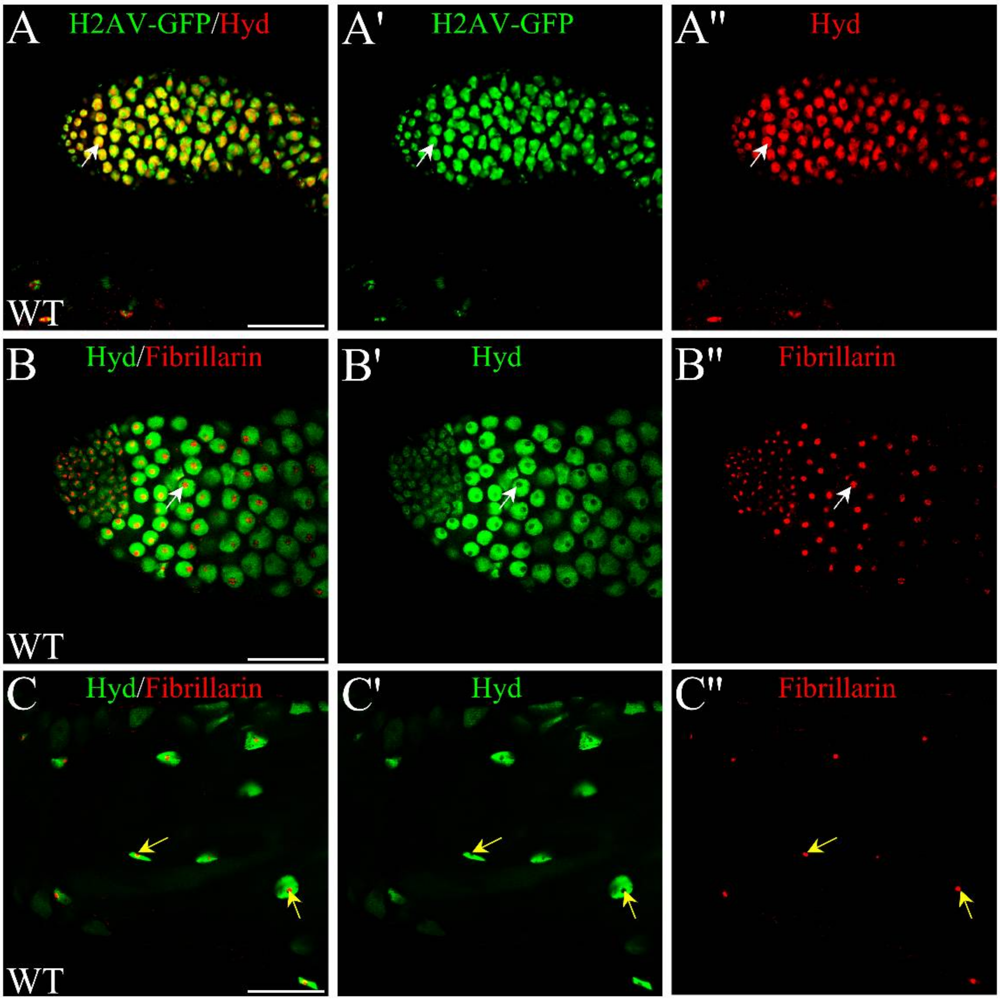

Supplementary Figure S1. Subcellular localization of Hyd in *Drosophila* testes. (**A-A''**) Hyd co-localizes with the nuclear marker H2AV-GFP. (**B-C''**) Hyd is excluded from Fibrillarin-positive nucleoli in both germline (white arrow) and late cyst cells (yellow arrow). Scale bar, 50 μm.

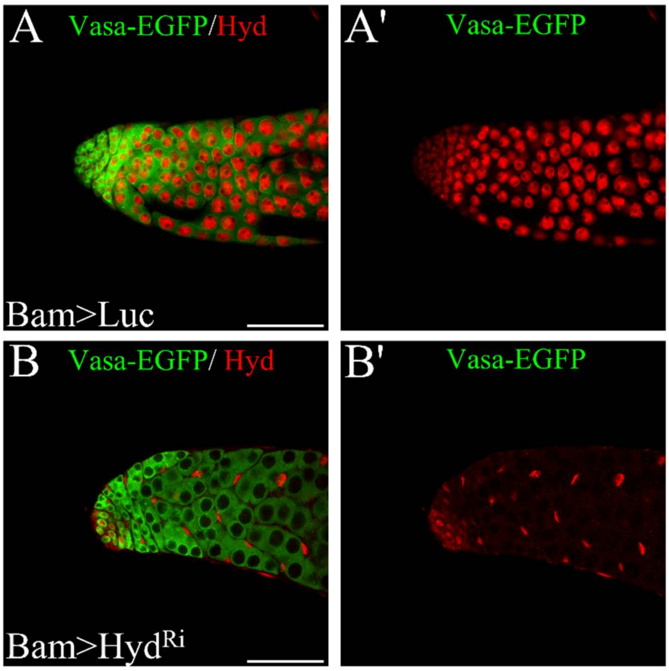

Supplementary Figure S2. Hyd knockdown efficiently depletes Hyd protein in the testis. (**A-A'**) In control testes, Hyd is highly expressed in spermatogonia. (**B-B'**) Depletion of Hyd driven by Bam-gal4-vp16 (Bam>Hyd^{Ri}) results in a significant reduction of Hyd expression, confirming the efficiency of the RNAi line. Vasa-EGFP label germlines, Scale bar, 50 μm.

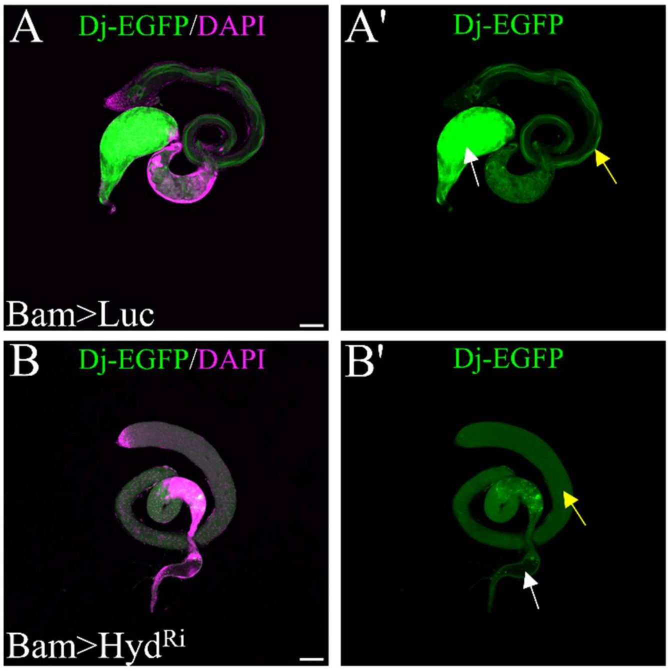

Supplementary Figure S3. Hyd knockdown depletes elongated spermatids and mature sperm. (**A-A'**) Dj-GFP labeling reveals the presence of elongated spermatid bundles (yellow arrow) and mature sperm (white arrow) in control testes. (**B-B'**) Both elongated spermatid bundles and mature sperm are absent in *Bam>Hyd-RNAi* testes. Scale bar, 50 µm.

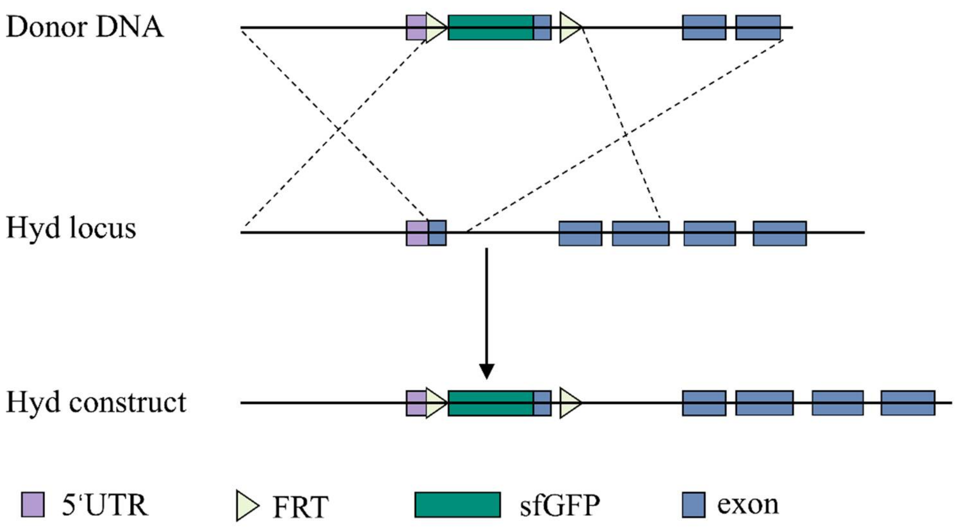

Supplementary Figure S4. Strategy for CRISPR/Cas9-mediated generation of the *GFP-Hyd* knock-in allele. Schematic of the strategy for replacing the endogenous *hyd* locus with a donor construct containing FRT sites, sfGFP, and the first exon of *hyd*.

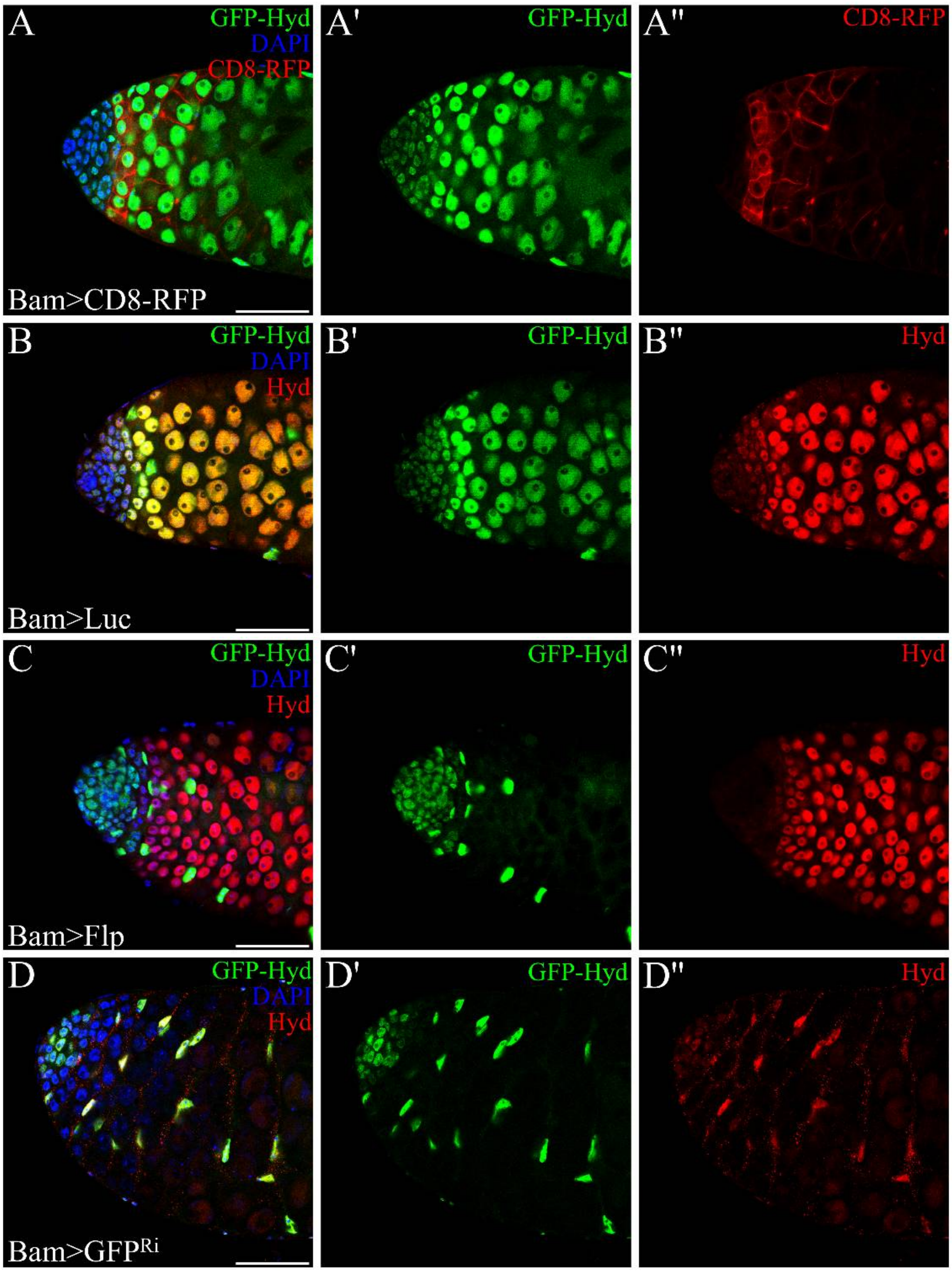

Supplementary Figure S5. Hyd inactivation by FLP excision and GFP-RNAi knockdown. (**A-A'**) *Bam-Gal4-VP16* drives CD8-RFP expression primarily in 4-16 cell germlines. (**B-B'**) GFP-Hyd signal co-localizes with anti-Hyd antibody staining in control testes. (**C-C'**) FLP-mediated excision under *Bam-Gal4-VP16* removes sfGFP but does not abolish Hyd protein expression in spermatogonia. (**D-D'**) *Bam>GFP-RNAi* efficiently knocks down both sfGFP and Hyd in the *GFP-Hyd* background. Scale bar, 50 μm.

Supplementary primer sequence:

Table S1. Primer sequences used for plasmid construction and verification as follows.

Primer Name	Sequence (5' → 3')	Purpose
hyd-gRNA-F:	CTTCGAATTACGCACATGTGAACG	
hyd-gRNA-R:	AAACCGTTCACATGTGCGTAATTC	
BamH Ⅰ -hyd-5Arm-F:	ttaGGATCCTTCCCTATTGCGGTAGTGATGGT	5′-Arm construction
KpnI-hyd-5Arm-R:	ttaGGTACCCATGAAGTTCCTATACTTTCTAGAGAATAGGAACTTCTTTAATTTTTCAACTATTCATGTACACAG	
BglII-hyd-3Arm-F1:	ttaAGATCTGTTTCCATGCAATTTGTTTTGCAAC	5′-Arm construction
Hyd-3Arm-S1:	gttCCTATACTTTCTAGAGAATAGGAACTTCTGCGTTCACATGTGCGTAATTG	
Hyd-3Arm-F2:	GTTCCTATTCtctagaaaGtATAGGAACTTCTGTGTAATTGTGTACGTACGG	
XhoI-Hyd-3Arm-S2:	ttaCTCGAGCTCTTGCCCTGTAGAACCACCTC	
sfGFP-F:	GATGACGGCACCTACAAGACC	sfGFP identification
sfGFP-R:	CTCTGGGTGCTCAGGTAGTGG	
GFP-Hyd-F:	CCGATAAGAATGGACGCATTCGTG	GFP-Hyd tagin fly identification
GFP-Hyd-R:	TGCCATGTGCTGAAAGAGCG	
FRT sequence:	GAAGTTCCTATTCtctagaaaGtATAGGAACTTC	
